# Comparative Genomics of *Vibrio cholerae* from Haiti, Asia, and Africa

**DOI:** 10.3201/eid1711.110794

**Published:** 2011-11

**Authors:** Aleisha R. Reimer, Gary Van Domselaar, Steven Stroika, Matthew Walker, Heather Kent, Cheryl Tarr, Deborah Talkington, Lori Rowe, Melissa Olsen-Rasmussen, Michael Frace, Scott Sammons, Georges Anicet Dahourou, Jacques Boncy, Anthony M. Smith, Philip Mabon, Aaron Petkau, Morag Graham, Matthew W. Gilmour, Peter Gerner-Smidt, the V. cholerae Outbreak Genomics Task Force

**Affiliations:** Author affiliations: Public Health Agency of Canada, Winnipeg, Manitoba, Canada (A.R. Reimer, G. Van Domselaar, M. Walker, H. Kent, P. Mabon, A. Petkau, M. Graham, M.W. Gilmour); Centers for Disease Control and Prevention, Atlanta, Georgia, USA (S. Stroika, C. Tarr, D. Talkington, L. Rowe, M. Olsen-Rasmussen, M. Frace, S. Sammons, P. Gerner-Smidt); Centers for Disease Control and Prevention, Port-au-Prince, Haiti (G.A. Dahourou); Ministry of Public Health and Population, Port-au-Prince (J. Boncy); National Institute for Communicable Diseases, Johannesburg, South Africa (A.M. Smith)

**Keywords:** Vibrio cholerae, cholera, bacteria, genomics, whole-genome sequencing, public health, outbreak, epidemiology, Haiti, Asia, Africa, travel

## Abstract

Cholera was absent from the island of Hispaniola at least a century before an outbreak that began in Haiti in the fall of 2010. Pulsed-field gel electrophoresis (PFGE) analysis of clinical isolates from the Haiti outbreak and recent global travelers returning to the United States showed indistinguishable PFGE fingerprints. To better explore the genetic ancestry of the Haiti outbreak strain, we acquired 23 whole-genome *Vibrio* *cholerae* sequences: 9 isolates obtained in Haiti or the Dominican Republic, 12 PFGE pattern-matched isolates linked to Asia or Africa, and 2 nonmatched outliers from the Western Hemisphere. Phylogenies for whole-genome sequences and core genome single-nucleotide polymorphisms showed that the Haiti outbreak strain is genetically related to strains originating in India and Cameroon. However, because no identical genetic match was found among sequenced contemporary isolates, a definitive genetic origin for the outbreak in Haiti remains speculative.

## Abstract 2

A strain from Haiti shares genetic ancestry with those from Asia and Africa.

The current (seventh) cholera pandemic was caused by serogroup O1 El Tor biotypes of *Vibrio cholerae*. This biotype first emerged on the Indonesian island of Sulawesi in 1961, then subsequently spread throughout Asia and Africa, where endemic and epidemic disease persists today (*1*,*2*). Seventh cholera pandemic biotypes were introduced into Peru in 1991 and subsequently spread across South and Central America, but these biotypes never reached the island of Hispaniola. Recent endemic and epidemic cases in Asia and Africa are increasingly attributed to genetically atypical El Tor variants that share characteristics of classical and El Tor strains (*1*,*3*,*4*).

After the 2010 earthquake in Haiti, an outbreak of cholera emerged that resulted in >385,000 infections and 5,800 deaths as of July 7, 2011 (*5*). The outbreak strain quickly spread to the neighboring Dominican Republic and globally as travelers returned home from affected regions (*6*,*7*). Concurrent cholera cases in the United States, linked by travel to cholera-endemic regions in Asia and Africa, were identified by national surveillance activities of PulseNet USA (Centers for Disease Control and Prevention [CDC], Atlanta, GA, USA.)

Serotyping, biotyping, and pulsed-field gel electrophoresis (PFGE) fingerprinting investigations suggested that the travel-associated cases could be genetically related to the Haiti outbreak strain (*8*). Because of the historical absence of cholera in Haiti before the 2010 earthquake, speculation abounds that the outbreak strain was imported into Haiti. Although clonality of the Haiti outbreak strain has been inferred by phenotypic characterization and genotypic subtyping, thereby supporting a single foreign source hypothesis (*6*,*8*), definitive evidence, e.g., by whole-genome sequencing for the genetic ancestry of the Haitian strain, is lacking.

Preliminary comparative analysis of whole-genome sequences from two 2010 Haiti outbreak isolates with genomes from historical cholera cases resulted in speculation that the outbreak originated in southern Asia (*9*). However, this study lacked recent, globally distributed cholera case isolates and particularly lacked studied genomes from Africa, to which cholera is endemic. We selected contemporary *V. cholerae* isolates from clinical infections, attributed to geographically distinct locations and sharing PFGE fingerprints with Haiti outbreak strains, from the PulseNet USA database for comparative whole-genome analysis. Although detailed epidemiologic investigations are essential for unequivocally attributing geographic origin(s) and means of cholera introduction into Haiti, genome sequences of these 23 contemporary isolates showed details related to genetic content and diversity that were otherwise missed with lower-resolution PFGE subtyping, thereby providing useful genetic ancestry information for interpreting the outbreak in Haiti.

## Methods

### Patients and Isolates

*V. cholerae* isolates and travel histories from cholera case-patients in the United States were referred to CDC. A strain from an outbreak in Cameroon in 2010, isolated from a specimen received at CDC, and an isolate from South Africa likely linked to an outbreak in Zimbabwe in 2009 were also included in this study (*10*). Isolates C6706 and 3569–08 were acquired during the outbreak in Latin America in 1991 and from the US Gulf Coast in 2008, respectively. All strains were characterized as *V. cholerae* O1 on the basis of standard biochemical, cholera toxin, and serologic testing performed as described (*11*,*12*). PFGE was performed according to the PulseNet standardized protocol with restriction enzymes *Sfi*I and *Not*I; PFGE patterns were designated by using BioNumerics version 5.10 (Applied Maths Inc., Sint-Martins-Latem, Belgium) and compared by unweighted pair group method with arithmetic mean analysis (DICE coefficient 1.5% tolerance and optimization). Strain designations and other information are shown in Table 1.

**Table 1:** Characteristics of *Vibrio cholerae* isolates from Haiti, Asia, Africa, and the United States*

Isolate	Serotype	*ctxB* allele†	PFGE patterns		Date of collection	Origin of infection	Comment or GenBank accession no. (reference)
			*Sfi*I	*Not*I			
2010EL-1961	Ogawa	B-7	KZGS12.0088	KZGN11.0092	2010 Oct 17	Haiti	Earliest Haiti outbreak case
2010EL-1786	Ogawa	B-7	KZGS12.0088	KZGN11.0092	2010	Artibonite, Haiti	None
2010EL-1792	Ogawa	B-7	KZGS12.0088	KZGN11.0092	2010	Artibonite, Haiti	None
2010EL-1798	Ogawa	B-7	KZGS12.0088	KZGN11.0092	2010	Haiti	None
2010EL-2010N	Ogawa	B-7	KZGS12.0160	KZGN11.0134	2010	Haiti	Nonhemolytic
2010EL-2010H	Ogawa	B-7	KZGS12.0088	KZGN11.0092	2010	Haiti	Hemolytic
2011EL-1089	Ogawa	B-7	KZGS12.0088	KZGN11.0092	2010 Nov 27	South Department, Haiti	None
2011EL-1133	Ogawa	B-7	KZGS12.0088	KZGN11.0092	2011 Jan 26	Northwest Department, Haiti	Travel associated
2011V-1021	Ogawa	B-7	KZGS12.0088	KZGN11.0092	2011	Dominican Republic	Travel associated
2009V-1085	Ogawa	B-7	KZGS12.0088	KZGN11.0092	2009	Sri Lanka/India	Travel associated
2009V-1096	Inaba	B-7	KZGS12.0088	KZGN11.0092	2009	India	Travel associated
2010EL-1749	Ogawa	B-7	KZGS12.0088	KZGN11.0092	2010	Cameroon	Outbreak
2009V-1131	Ogawa	B-7	KZGS12.0088	KZGN11.0092	2009	India	Travel associated
3554–08	Ogawa	B-7	KZGS12.0088	KZGN11.0092	2008	Nepal	Travel associated
2011EL-1137	Ogawa	B-1	KZGS12.0089	KZGN11.0092	2009	South Africa	Outbreak
2009V-1046	Ogawa	B-1	KZGS12.0088	KZGN11.0092	2009	Pakistan	Travel associated
2009V-1116	Ogawa	B-1	KZGS12.0088	KZGN11.0092	2009	Pakistan	Travel associated
2010V-1014	Ogawa	B-1	KZGS12.0088	KZGN11.0092	2010	Pakistan	Travel associated
3582–05	Inaba	B-1	KZGS12.0088	KZGN11.0092	2005	Pakistan	Travel associated
3500–05	Inaba	B-1	KZGS12.0088	KZGN11.0092	2005	India	Travel associated
3546–06	Inaba	B-1	KZGS12.0088	KZGN11.0092	2006	India	Travel associated
3569–08	Inaba	B-1	KZGS12.0055	KZGN11.0029	2008	US Gulf Coast	Environmental isolate
C6706	Inaba	B-3	KZGS12.0114	KZGN11.0033	1991	Peru	Latin America outbreak
CIRS101‡	Inaba	B-1	Unknown	Unknown	2002	Dhaka, Bangladesh	NZ_ACVW00000000 (*4*)
MJ-1236‡	Inaba	B-1	Unknown	Unknown	1994	Matlab, Bangladesh	NC_012667, NC_012668 (*4*)
O395‡	Ogawa	B-1	Unknown	Unknown	1965	India	NC_009456, NC_009457 (*1*)
N16961‡	Inaba	B-3	Unknown	Unknown	1970s	Bangladesh	NC_002505, NC_002506 (*13*)
M66–2‡	Unknown	§	Unknown	Unknown	1937	Makassar, Indonesia	NC_012578, NC_012580 (*1*)

*ctxB, cholera toxin subunit B; PFGE, pulsed-field gel electrophoresis. †B-7, classical allele, Orissa variant; B-1, classical allele; B-3, El Tor allele (*2*) ‡These isolates have been sequenced by other investigators, and sequences have been deposited in GenBank. PFGE was not performed on these isolates. §This isolate does not contain *ctxB*.

### Whole-Genome Data Acquisition, Assembly, and Annotation

Single-end pyrosequencing reads (GS FLX-Titanium; Roche Diagnostics, Indianapolis, IN, USA) and single-end 36-bp or 76-bp Illumina reads (GAIIe sequencer; Illumina, San Diego, CA, USA) were acquired and yielded >99% genome coverage and 32× and 240× average coverage depths, respectively (Table 2). Pyrosequencing reads were first assembled de novo by using Newbler version 2.5.3 (Roche Diagnostics). To correct potential base-calling errors attributed to homopolymers, Illumina GAIIe reads (average 14 million reads/genome) were mapped to the Newbler contigs by using CLC Genomics Workbench version 4.5 (www.clcbio.com/index.php?id=1042) and yielded an average combined coverage depth of 270× per genome.

**Table 2:** Next-generation sequence average coverage and number of mapped reads for *Vibrio cholerae* isolates from Haiti, Asia, and Africa

Isolate	No. mapped Illumina reads*	Average Illumina coverage*	No. 454 aligned reads†	Average 454 coverage†
2009V-1046	12,100,798	167.5	288,870	28
2009V-1085	13,679,291	187.8	365,484	33
2009V-1096	14,818,679	205.2	649,798	60
2009V-1116	13,486,955	181.8	264,833	23
2009V-1131	1,370,5972	185.9	273,608	24
2010EL-1749	16,654,195	189.7	735,029	51
2010EL-1786	26,312,006	343.8	216,539	17
2010EL-1792	23,073,959	295.9	239,940	19
2010EL-1798	27,914,201	369.9	270,493	21
2010V-1014	15,247,545	195.5	501,200	44
3500–05	10,962,437	268.6	279,246	27
3546–06	14,625,431	331.0	238,176	22
3569–08	15,920,777	201.4	228,302	18
3582–05	12,181,066	302.0	621,605	62
C6706	15,578,468	349.4	363,226	35
2010EL-1961	9,077,044	229.0	415,643	40
2011EL-1089	10,841,303	263.2	194,828	17
2011EL-1133	12,544,418	283.8	112,039	10
2011EL-1137	11,703,624	285.2	505,482	48
2011EL-2010N	12,178,627	323.5	409,268	41
2011V-1021	11,274,787	282.8	213,312	20
2010EL-2010H	11,366,854	291.4	422,937	40
3554–08	16,149,256	373.9	498,131	45

*Determined by using GAIIe Sequencer; Illumina, San Diego, CA, USA. †Determined by using 454 Sequencer; 454 Life Sciences, Branford, CT, USA.

Both chromosomes of Haiti outbreak isolate 2010EL-1786 were sequenced to full closure by using PCR and Sanger sequence-based bridging of contigs and a fosmid library of templates. Optical mapping also supported the contig ordering derived for 2010EL-1786. For all remaining isolates, Illumina-supplemented, homopolymer-corrected, Newbler-assembled contigs were prepared as pseudogenomes by first linking contigs with a linker sequence containing stop codons in all 6 translation reading frames. These high-coverage pseudogenomes were used for downstream analyses. Identification of coding sequences was achieved by using Glimmer3 (*14*). Genome annotation was achieved by using an automated, in-house, modified version of GenDB version 2.2 (*15*) and manual curation for regions of interest.

### Comparative Genomics

#### Whole-Genome Alignment and Core Genome Phylogeny

Whole-genome alignments of all study isolates and 5 available reference *V. cholerae* genomes (Table 1) were performed by using Progressive Mauve (*16*) and visualized by using PhyML 3.0 (*17*). To determine vertical inheritance patterns, study genomes were analyzed with historical *V. cholerae* genomes (isolates M66–2, MJ-1236, CIRS101, and N16961) by using phylogenetic analysis of high-quality single-nucleotide polymorphisms (hqSNPs) contained in core genes. Coding region predictions were analyzed by using parallelized BLASTn (http://blast.ncbi.nlm.gov/Blast.cgi) to identify highly similar orthologs in all strains. Highly similar orthologs were defined as those containing a high-scoring segment pair >400 bp and identity >97%. Each orthologous loci set was multiply aligned by using ClustalW (*18*). Multiple alignments were manually inspected to remove erroneously aligned regions; indel-associated SNPs and loci containing >30 SNPs were also excluded. Each SNP column from each multiple nucleotide alignment was analyzed for hqSNPs, defined as those containing no gaps or ambiguous basecalls, and having an adjusted quality score >90 (of a maximum score of 93). A total of 4,376 hqSNPs were identified from 632 orthologous loci and extracted from the alignments to prepare a compressed pseudoalignment composed of hqSNPs (Technical Appendix 1). This pseudoalignment was used to build a maximum-likelihood phylogenetic tree by using PhyML 3.0 (*17*). Branch confidences were estimated by using the approximate likelihood-ratio test (*19*).

#### BLAST Atlases

A circular BLAST atlas was generated for each chromosome by using Haiti isolate 2010EL-1786 as mapping reference. Glimmer3 was used to predict coding sequences contained on pseudogenomes for the remaining isolates sequenced in this study and for 4 available genomes (*14*). Reference isolate 2010EL-1786 was mapped against the resulting translated coding sequences by using BLASTx with a percentage identity cutoff value of 70% and an expected cutoff value of 1 × 10^–10^ for high-scoring segment pairs >100 aa. The results were visualized by using GView (*20*). Sequence accession numbers are shown in Table 1.

## Results

### *Sfi*I and *Not*I PFGE Patterns of Recent Global Cholera Isolates

Nine *V. cholerae* isolates directly associated with the outbreak on Hispaniola were examined, 7 of which had indistinguishable *Sfi*I and *Not*I PFGE patterns designated PulseNet USA patterns KZGS12.0088 and KZGN11.0092, respectively (Table 1). Also sequenced were a hemolytic variant and a nonhemolytic variant that harbored a minor variation of the main Haiti outbreak PFGE pattern and were derived from an isolate from 1 patient in Haiti (Table 1). Twelve contemporary *V. cholerae* isolates from global sources with matched PFGE fingerprints were also sequenced. Infections for these 12 contemporary isolates originated (by documented patient travel) from regions of Pakistan, India, or Nepal. Two additional isolates were from patients in outbreaks in Cameroon and South Africa likely connected to the cholera outbreak in Zimbabwe in 2009 (*21*). Although all sequenced clinical isolates were serogroup O1, Inaba and Ogawa serotypes were observed among PFGE pattern-matched isolates (Table 1). All strains were biotype El Tor and all produced cholera toxin.

### Phylogenetics of Strains

**Figure 1:**
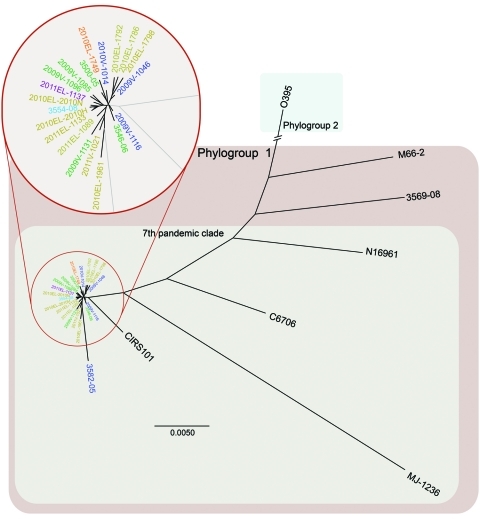
Whole-genome neighbor-joining tree of *Vibrio cholerae* isolate from cholera outbreak in Haiti, fall 2010; concurrent clinical isolates with pulsed-field gel electrophoresis pattern-matched combinations; reference isolates sequenced in this study; and available reference sequences. Sequence alignments of quality draft or complete genomes were performed by using Progressive Mauve (*16*) and visualized by using PhyML version 3.0 (*17*). Whole-genome relationship of Haiti isolates with closest genetic relatives is shown in the inset. Scale bar indicates nucleotides substitutions per site.

Haiti outbreak isolates and 12 global PFGE pattern-matched *V. cholerae* isolates belong to phylogroup 1 of the seventh pandemic clade. The phylogenetic tree based on whole-genome sequencing showed clustering of the 9 Hispaniola isolates (8 from Haiti and 1 related isolate from the Dominican Republic) with 12 other PFGE pattern-matched isolates. All 21 isolates were in 1 cluster relative to non-PFGE–pattern-matched outliers (Figure 1). When compared with historical reference genomes, the closest ancestors for Haiti genome sequences (2010–2011; derived herein) were isolates CIRS101 from Dhaka, Bangladesh (2002) and MJ-1236 from Matlab, Bangladesh (1994). These data confirm the genetic relatedness also inferred by PFGE subtyping and further support inclusion of the Haiti outbreak isolates in phylogroup 1 of the seventh pandemic clade (Figure 1). The whole-genome sequencing dataset showed that additional underlying genetic diversity was present across PFGE pattern-matched isolates (including 9 isolated from Hispaniola) not observed by PFGE subtyping.

### Common Mobile Elements and Genes of Haiti Outbreak Strain and PFGE Pattern-matched Isolates

**Figure 2:**
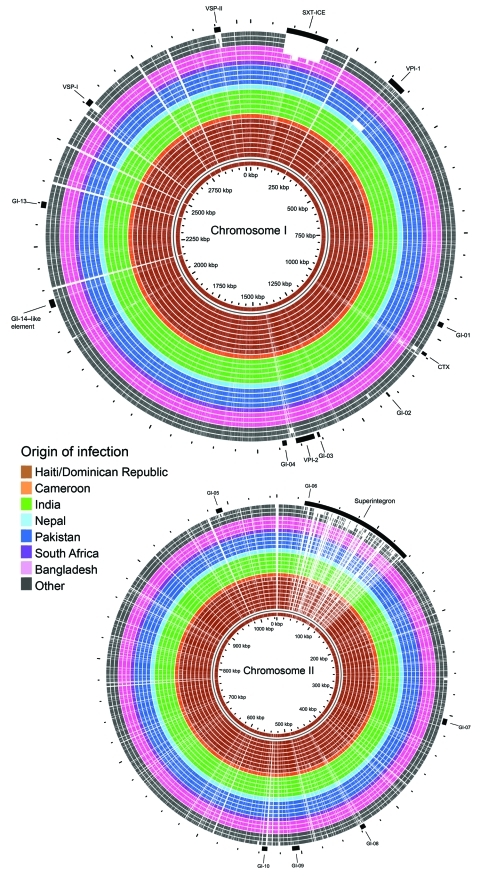
BLAST (http://blast.ncbi.nlm.gov/Blast.cgi) atlas of predicted protein homologies mapped against the closed genome of Haiti *Vibrio cholerae* outbreak type isolate 2010EL-1786, fall 2010. Full color saturation represents 100% sequence homology, and gaps indicate regions of divergence. Gaps in the innermost (red) circle for reference isolate 2010EL-1786 represent gaps between coding sequences, not genetic diversity. A) Chromosome I; B) chromosome II. From center: Haiti/Dominican Republic isolates 2010EL-1786, 2010EL-1961, 2011EL-1089, 2010EL-2010N, 2010EL-2010H, 2011V-1021, 2010EL-1798, 2010EL-1792, and 2011EL-1133; Cameroon isolate 2010EL-1749; India isolates 2009V-1085, 2009V-1096, 2009V-1131, 3546–06, and 3500–05; Nepal isolate 3554–08; Pakistan isolates 3582–05, 2009V-1046, 2010-V1014, and 2009V-1116; South Africa isolate 2011EL-1137; Bangladesh isolates CIRS101, MJ-1236 and N16961; and other isolates C6706, M66–2, and 3569–08.

*V. cholerae* macrodiversity is commonly attributed to presence or absence of mobile genetic elements (*22*). The contiguous genome derived for Haiti isolate 2010EL-1786 was used as the outbreak type strain and harbored 2 circular chromosomes of 3.03 Mbp (chromosome I) and 1.05 Mbp (chromosome II), which encoded 2,920 and 1,051 predicted coding sequences, respectively. Pairwise comparisons of all coding sequences from each study genome with all coding sequences from reference isolate 2010EL-1786 (all vs. all comparison) showed congruent gene content and low overall diversity on larger chromosome I (Figure 2). One noteworthy exception was the absence of *Vibrio* pathogenicity island 1 in the 2005 isolate 3582–05 from Pakistan. This island contains essential cholera virulence factors, including the *tcp* gene cluster, which encodes toxin-coregulated pilus involved in *V. cholerae* colonization of the human intestine and necessary for horizontal transfer of the cholera toxin bacteriophage. This finding was the only macroscopic difference observed between isolate 3582–05 and PFGE matches. All Haiti outbreak and PFGE pattern-matched isolates contain an integrated conjugative element belonging to the SXT/R391 family (SXT-ICE) that carries genes conferring antimicrobial drug resistance. No macroscopic differences were observed in SXT-ICE among Haiti outbreak and PFGE pattern-matched isolates (Figure 2; Technical Appendix 2 Figure 1, panel A).

Smaller chromosome II was more content variable and divergent across study strains. These findings were largely attributable to the hypervariable superintegron region, an ≈120-kb gene capture system predominantly encoding hypothetical proteins (Figure 2; Technical Appendix 2 Figure 1, panel B) (*13*). Gene polymorphisms observed in the 9 sequenced isolates from Hispaniola also localized primarily within the superintegron region. Despite these observed differences, no major deletions in the superintegron were observed among PFGE pattern-matched isolates (Figure 2; Technical Appendix 2 Figure 1). Thus, phylogeny derived from *V. cholerae* whole-genome sequencing (Figure 1) showed genetic diversity within PFGE pattern-matched isolates. However, binary (present or absent) gene content assessment failed to pinpoint extensive contiguous diversity outside the superintegron region.

### Shared Ancestry between Isolates from Haiti, India, and Cameroon

A core genome phylogeny was constructed on the basis of 4,376 hqSNPs found within 632 orthologous core genes (0.81 Mbp) that were universally present in all 27 study and reference genomes (Technical Appendix 1; Technical Appendix 2 Figure 2). Among 9 sequences from Hispaniola isolates, 0–2 SNPs were observed (Technical Appendix 2 Figure 2). Hispaniola isolates differed from PFGE pattern-matched genomes from other locations by 4–25 SNPs, and genomes with nonmatched PFGE patterns differed from the outbreak isolates by 13–3,361 SNPs. Notably, phylogeny based on hqSNPs showed clustering of the Haiti strain with 3 epidemiologically unrelated clinical isolates, which represented isolates from 2 travelers from the United States to India in 2009 and a patient in Cameroon in 2010. Isolates 2009V-1085 (India, 2009), 2009V-1096 (India, 2009), and 2010EL-1749 (Cameroon, 2010) were most related to the Haiti isolates. These 3 isolates had 4–7 core hqSNPs when compared with the outbreak strain, and the derived sequence for a 2008 clinical isolate from Nepal differed from outbreak isolates by 7–8 core hqSNPs (Technical Appendix 1; Technical Appendix 2 Figure 2).

**Figure 3:**
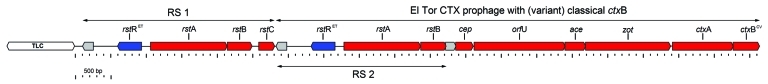
Genetic structure of cholera toxin (CTX) prophage and associated elements in Haiti cholera outbreak *Vibrio cholerae* isolate 2010EL-1786, fall 2010. The toxin-linked cryptic (TLC) element is not drawn to scale. Black arrows indicate the direction of transcription for each coding region. Red, forward transcription; blue, reverse transcription; gray, predicted open reading frame with no experimental evidence.

Conversely, historical isolates (1970–2005) from Pakistan, Bangladesh, the US Gulf Coast, and South America, and recent clinical isolates (2009–2010) from cases linked to Pakistan or South Africa independently clustered away from Haiti outbreak isolates (Technical Appendix 2 Figure 2). Clade analysis of outbreak isolates and highly related isolates 2009V-1085, 2009V-1096, and 2010EL-1749, identified 25 hqSNPs in 24 conserved loci that distinguish members of this clade (Technical Appendix 2 Figure 3; Table A1). Resulting distances suggest that the outbreak isolates have a closer genetic relationship with 2009V-1085 and 2009V-1096 from India (7–10 hqSNPs) than with 2010EL-1749 from Cameroon (10–13 hqSNPs).

### Comparison of Haiti Outbreak Genomes

Across the 18 described hypervariable *V. cholerae* mobile genetic elements sequences (representing >300 kb of the total genome), no macroscopic differences were observed among the 9 Hispaniola isolate sequences (Figure 2; Technical Appendix 2 Figure 1), and as stated, only 2 hqSNPs were identified in the core genome. Pairwise alignment of the complete genome of study reference 2010EL-1786 with available genome data for 2 sequenced Haiti 2010 outbreak isolates, designated H1 and H2 (*9*), showed only 3 polymorphisms across the entire genome. However, because the available H1 and H2 consensus sequences contain ambiguous basecalls, these nucleotides were excluded from our comparative analyses. Nonetheless, these data confirm the clonal nature of the Haiti outbreak strain.

### Structural and Alleleic Profiles of Isolates Carrying a Hybrid Cholera Toxin Prophage

Structure and allelic profiles of the CTXϕ prophage have been used for *V. cholerae* lineage analysis (*23*). Chromosome I of Haiti isolate 2010EL-1786 harbors 1 hybrid CTXϕ characterized by a 1-nt variant of the classical *ctxB* allele (*ctxB*-7) and El Tor *rstR* flanked by a toxin-linked cryptic element and El Tor–type RS1 element with an intact *rstC* locus (Figure 3). The SNP at *ctxB* codon19 results in replacement of the classical cholera toxin B histidine residue with asparagine, and this *ctxB*-7 allele was observed among all Hispaniola isolates (Table 1). Five of the 12 PFGE pattern-matched isolates from other locations (2008–2010) also shared this variant *ctxB* allele. The remaining 7 PFGE pattern-matched isolates encoded classical *ctxB* alleles.

## Discussion

Public health investigators use PFGE, the current standard technique for subtyping most bacterial enteric pathogens, to link patients infected with a particular pathogen to a specific infection source(s) by fingerprint matching to pathogens isolated from environmental samples. Whole-genome sequencing has recently emerged as an enhanced laboratory tool for high-resolution analysis of microbial diversity and has been successfully used to investigate bacterial disease outbreaks (*24*–*26*). Because whole-genome sequencing can provide pathogen genetic fingerprints at single-nucleotide resolution, it should revolutionize the diagnosis, surveillance, and control of microbial diseases.

For molecular epidemiologic investigations using whole-genome sequencing, an expansive number of isolates from an outbreak would ideally be selected to ensure broad coverage for possible genotype variants within that population that might otherwise be masked with lower-resolution typing methods. In addition, outlier isolates from different locations that are indistinguishable or related by several diverse subtyping methods should also be subjected to whole-genome sequencing to contextualize the diversity seen within the outbreak population and to find other clonal relationships In this study, a temporal and geographic distribution of outbreak isolates was selected to confirm clonality of the outbreak strain and to gain insight into the microevolution of *V. cholerae* during an outbreak. Additionally, minor PFGE and nonhemolytic variants observed among outbreak isolates were also sequenced to confirm their clonal relationships with isolates exhibiting the main outbreak pattern and phenotype.

The PulseNet USA database substantially contributed to this work by identifying genetically related (using PFGE typing) and epidemiologically relevant isolates for whole-genome sequencing analyses. Notably, one 2008 isolate from a traveler from the United States to Nepal was identified and included in this study, although we acknowledge that the evolutionary relationship of the Haiti strain to strain(s) circulating in Nepal during 2010 may not be ideally represented by this 2008 isolate. Microbial evolution will have occurred during 2008–2010, and global travel may have introduced additional strains into Nepal in the interim, such that the 2008 isolate from Nepal may differ substantially from a strain circulating in Nepal in 2010, the suggested progenitor of the outbreak strain. Unfortunately, 2010 isolates from Nepal were not available for analysis.

Also identified in the PulseNet USA database was 1 PFGE pattern-matched isolate from western Africa. The close genetic relationship of this isolate from Cameroon to the Haiti strain suggests that a potential link between western Africa and the Haiti outbreak cannot be ignored. Further studies on additional isolates from western Africa are required to confirm or refute this possibility. Similarity of whole-genome sequences for Haiti isolates, PFGE pattern-matched isolates, and other seventh pandemic strains confirmed the clonal nature of the 2010–2011 cholera outbreak strain and the close genetic relationships for the studied strains initially suggested by PFGE subtyping (Figure 1). Previous *V. cholerae* studies have reported that seventh pandemic strains are clonal, sharing near identical gene content on a highly related genome backbone but containing variable mobile genetic elements or gene cassettes (*27*). Despite dynamic horizontal gene transfer (*22*), we identified only a few nucleotide differences among mobile sequences of the 9 sequenced 2010–2011 outbreak-related Hispaniola isolates and the 12 recent PFGE pattern-matched clinical isolates (Figure 2).

Extensive recombination in *V. cholerae* genomes may confound evolutionary relationship analyses as strains and lineages undergo reassortment (*1*). However, base substitutions acquired horizontally as recombination segments generally occur with localized density (*28*). Although we cannot guarantee that recombinant segments were absent from the core genome phylogeny (Technical Appendix 2 Figure 2), the even spatial and genome-wide distribution of core genome hqSNPs suggests that they were vertically inherited. We have derived a useful phylogenetic approximation of isolate relatedness on the basis of hqSNPs, which supports shared ancestry for the Haiti outbreak isolates and 12 recent clinical isolates sharing PFGE patterns (Technical Appendix 2 Figure 2). Sequenced isolates from India and Cameroon (2009–2010) were shown to be the closest genetic relatives among the non-Hispaniola isolates (isolated in 1991–2010; this study) and 4 other available reference *V. cholerae* genomes (isolated in 1937–2002). The *ctxB* allele variant (*ctxB*-7) of the Haiti strain (and its genetic relatives) was first observed among isolates from a cholera outbreak in Orissa, India, in 2007 (*29*), but the *ctxB*-7 allele has since also been observed in isolates from southern Asia and more recently from western Africa (*8*,*30*).

The genetic makeup of the Haiti outbreak strain will likely have substantial public health implications for Haiti and other susceptible locations. Our reasoning is that the atypical O1 El Tor *V. cholerae* strains (CIRS101 and CIRS101-like variants) have already emerged as the predominant clone causing cholera in Asia and Africa and have displaced prototypical O1 El Tor strains (*3*,*4*,*29*). Unfortunately, atypical O1 El Tor *V. cholerae* strains appear to have retained the relative environmental fitness of their prototypical O1 El Tor ancestors while acquiring enhanced virulence traits, such as classical or hybrid CTX prophage and SXT-ICE (*4*). Thus, with higher relative fitness and virulent and antimicrobial drug–resistant phenotypes, the Haiti outbreak strain harbors infectivity and ecologic persistence advantages over other seventh pandemic strains. Consequently, the Haiti outbreak strain (or its genetic ancestor) may easily replace current El Tor *V. cholerae* strains circulating in the Western Hemisphere to become endemic (like other atypical El Tor strains) and will likely cause future outbreaks. Such dire predictions warrant enhanced epidemiologic surveillance and renewed priorities aimed at cholera prevention.

Absence of cholera in Haiti over the past century; the clonal nature of the outbreak strain; and a massive influx of international travelers, aid workers, and supplies after the 2010 earthquake suggest an outside infection source for the 2010–2011 outbreak. Our core genome phylogeny (Technical Appendix 2 Figure 2) suggests that the Haiti outbreak strain most likely derived from an ancestor related to isolates from within or near the Indian subcontinent. However, concurrent identification of a 2010 isolate from Cameroon as a close genetic relative of the Haiti outbreak strain illustrates that whole-genome sequencing on such a relatively small number (n = 27) of *V. cholerae* isolates is insufficient to exclude other plausible ancestral geographic locations.

Our study results are consistent with recent findings of Chin et al. (*9*), who concluded that two 2010 Haiti outbreak isolates shared ancestry with variant O1 El Tor strains isolated in Bangladesh in 2002 and 2008 and a more distant relationship with an isolate from an outbreak in Latin American in 1991. The vertical inheritance pattern of hqSNPs in our study provide unequivocal genetic evidence for introduction of the outbreak strain into Haiti from an external source as opposed to local aquatic emergence. However, the specific geographic source and mode of entry of the outbreak strain into Haiti cannot be proven by microbiological investigations. Only large-scale epidemiologic studies and microbiological data can provide conclusive evidence of how cholera was introduced into Haiti. This whole-genome sequencing study provides expanded evidence that variant O1 El Tor *V. cholerae* appeared in Haiti by importation and has generated a whole-genome sequencing dataset for future study.

## Supplementary Material

Technical Appendix 1 Conserved open reading frames among all Vibrio cholerae isolates and high quality single nucleotide polymorphisms (hqSNPs) used to estimate the evolutionary relationship between study isolates.

Technical Appendix 2 Contig data, reconstructed core genome phylogeny of Vibrio cholerae figures.

## Appendix

**Table A1:** Conserved open reading frames and hqSNPs used to refine evolutionary relationship between highly related outbreak *Vibrio cholerae* isolates and isolates 2009V-1085 (India), 2009V-1096 (India), and 2010EL-1749 (Cameroon)*

Locus ID†	Product	hqSNP, 5′→3′‡	Chromosome	Chromosome location§	Reference allele¶	Major allele#	Minor allele**	Minor allele strains††
**70**	**Transposase Tn3 family protein**	**CAAAAACAAG[G/T]TCACTCATCA**	**1**	**93944**	**T**	**T**	**G**	**2011V-1021**
307	K10937 accessory colonization factor AcfB	CTTGTTTCTA[A/G]TCGACCATGA	1	379769	A	A	G	2010EL-2010N
307	K10937 accessory colonization factor AcfB	TGTTTCTATT[C/G]GACCATGATA	1	379771	C	C	G	2010EL-2010N
612	Anthranilate synthase component II	CGGGCTGCAT[A/G]CCAGAGCTGC	1	724118	G	G	A	2009V-1096
644	Conserved hypothetical protein	TTATGCCAAT[C/T]CCTTATTCCT	1	763380	C	C	T	2010EL-1749
769	Conserved hypothetical protein	TTGAGCTACT[C/T]GCGAGTGAAA	1	916351	C	C	T	2010EL-1749
771	GGDEF family protein	CTCCGGAACT[C/T]ACCTTATTAC	1	919120	C	C	T	2009V-1096
1199	K00426 cytochrome bd-I oxidase subunit II	GCGTTATCTT[C/T]ACCGCAGGTT	1	1453325	T	T	C	2009V-1085, 2009V-1096, 2010EL-1749
1221	K00656 formate C-acetyltransferase	TTCATGGGTT[C/T]TGGCAACACA	1	1479808	C	C	T	2010EL-1749
1302	K08305 membrane-bound lytic murein transglycosylase B	TTGTGGGGGG[G/T]TGAAAGTAAT	1	1581661	T	T	G	2010EL-1749
1580	Outer membrane protein OmpH	GAAGTCGTTT[G/T]GCAAAAGATG	1	1876106	G	G	T	2009V-1085, 2009V-1096, 2010EL-1749
1641	Exodeoxyribonuclease V, 67-kDa subunit	CTCAAATTGT[A/G]TTGCGATAAC	1	1936753	G	G	A	2009V-1085, 2009V-1096, 2010EL-1749
1923	Ribosomal protein S12 methylthiotransferase	GGCTGCCTCG[A/G]CGCGTGAAGA	1	2259456	G	G	A	2009V-1096
**2052**	**Alkaline serine exoprotease A precursor**	**TTTTAAGCTT[A/G]CATTGTTTCG**	**1**	**2405353**	**A**	**A**	**G**	**2009V-1085, 2009V-1096, 2010EL-1749**
2142	Shikimate-5-dehydrogenase	TATTCATACG[A/C]TGTTTGCACG	1	2502464	C	C	A	2010EL-2010N
**2247**	**K01414 oligopeptidase A**	**AGAGCGCGGA[G/T]TGCCAAGCTT**	**1**	**2617657**	**T**	**T**	**G**	**2010EL-1749**
**2292**	**O-antigen ligase**	**TGTAAGAAAA[A/T]TAAAATTTAA**	**1**	**2667285**	**T**	**A**	**T**	**2010EL-1798**
2453	Conserved hypothetical protein	ATCATGCAAC[A/G]AGCCAACTAT	1	2846497	A	G	A	2009V-1085
2475	Conserved hypothetical protein	TTGGAAAAGG[G/T]GATTTCCGAT	1	2874265	T	T	G	2010EL-1961
2539	Erythrose 4-phosphate dehydrogenase	TCGACAACAC[C/T]CATCTATGGC	1	2931479	T	T	C	2010EL-1749
143	Pyruvate:ferredoxin (flavodoxin) oxidoreductase	TATTGGATCG[C/T]ACCAAAGAGC	2	168453	C	C	T	2011V-1021
**167**	**GGDEF family protein**	**AATTCCACCA[G/T]GCTTGAACTC**	**2**	**199181**	**G**	**T**	**G**	**2009V-1085**
701	Transcriptional regulator CdgA	TTAGAACGCC[A/C]CCGCCGCAGC	2	856230	C	A	C	2010EL-1786
740	K11891 type VI secretion system protein ImpL	GCAGAGGCCG[A/G]CCAACCCATT	2	908788	G	G	A	2009V-1085
763	TagA-related protein	TGGAGTCCGG[G/T]GGGGTGGGAT	2	938668	G	G	T	2010EL-1798

*hqSNP, high-quality single-nucleotide polymorphisms; ID, identification. SNPs in **boldface** are unique to this clade, i.e., they are not represented in the all strain core genome hqSNO set (Technical Appendix 1). †From reference strain 2010EL-1786. ‡hqSNP and flanking sequences are reported in the coding direction for each locus. §Chromosomal coordinate for the hqSNP is taken from the direct strand of reference strain 2010EL-1786. ¶Refers to allele harbored by reference strain 2010EL-1786. #Refers to most abundant allele in strains being compared. **Refers to least abundant allele in the strains being compared. ††Refers to strains harboring least abundant allele being compared.

## References

[1] Feng L, Reeves PR, Lan R, Ren Y, Gao C, Zhou Z, A recalibrated molecular clock and independent origins for the cholera pandemic clones. PLoS ONE. 2008;3:e4053. 10.1371/journal.pone.0004053 19115014 PMC2605724

[2] Safa A, Nair GB, Kong RY. Evolution of new variants of *Vibrio cholerae* O1. Trends Microbiol. 2010;18:46–54. 10.1016/j.tim.2009.10.003 19942436

[3] Nguyen BM, Lee JH, Cuong NT, Choi SY, Hien NT, Anh DD, Cholera outbreaks caused by an altered *Vibrio cholerae* O1 El Tor biotype strain producing classical cholera toxin B in Vietnam in 2007 to 2008. J Clin Microbiol. 2009;47:1568–71. 10.1128/JCM.02040-08 19297603 PMC2681878

[4] Grim CJ, Hasan NA, Taviani E, Haley B, Chun J, Brettin TS, Genome sequence of hybrid *Vibrio cholerae* O1 MJ-1236, B-33, and CIRS101 and comparative genomics with *V. cholerae.* J Bacteriol. 2010;192:3524–33. 10.1128/JB.00040-10 20348258 PMC2897672

[5] Pan American Health Organization. Cholera and post-earthquake response in Haiti, 2011 Jul 25 [cited 2011 Aug 6]. http://www.who.int/hac/crises/hti/sitreps/haiti_health_cluster_bulletin_25july2011.pdf.

[6] Centers for Disease Control and Prevention. Update on cholera—Haiti, Dominican Republic, and Florida, 2010. MMWR Morb Mortal Wkly Rep. 2010;59:1637–41. 21178947

[7] Gilmour MW, Martel-Laferrière V, Lévesque S, Gaudreau C, Bekal S, Nadon C, *Vibrio cholerae* in traveler from Haiti to Canada. Emerg Infect Dis. 2011;17:1124–5. 21749787 10.3201/eid1706.110161 PMC3358221

[8] Talkington D, Bopp C, Tarr C, Parsons MB, Dahourou G, Freeman M, Characterization of toxigenic *Vibrio cholerae* from Haiti, 2010–2011. Emerg Infect Dis. 2011;17:2122–9. 10.3201/eid1711.110805 PMC3310580 22099116

[9] Chin CS, Sorenson J, Harris JB, Robins WP, Charles RC, Jean-Charles RR, The origin of the Haitian cholera outbreak strain. N Engl J Med. 2011;364:33–42. 10.1056/NEJMoa1012928 21142692 PMC3030187

[10] Archer BN, Cengimbo A, De Jong GM, Keddy KH, Smith AM, Sooka A, Cholera outbreak in South Africa: preliminary descriptive epidemiology on laboratory-confirmed cases, 15 November 2008 to 30 April 2009. Communicable Diseases Surveillance Bulletin. 2009;7:3–8.

[11] Sakazaki R, Shimada T. Serovars of *Vibrio cholerae* identified during 1970–1975. Jpn J Med Sci Biol. 1977;30:279–82. 599729 10.7883/yoken1952.30.279

[12] Sakazaki R, Tamura K, Gomez CZ, Sen R. Serological studies on the cholera group of vibrios. Jpn J Med Sci Biol. 1970;23:13–20. 5310994 10.7883/yoken1952.23.13

[13] Heidelberg JF, Eisen JA, Nelson WC, Clayton RA, Gwinn ML, Dodson RJ, DNA sequence of both chromosomes of the cholera pathogen *Vibrio cholerae.* Nature. 2000;406:477–83. 10.1038/35020000 10952301 PMC8288016

[14] Delcher AL, Bratke KA, Powers EC, Salzberg SL. Identifying bacterial genes and endosymbiont DNA with Glimmer. Bioinformatics. 2007;23:673–9. 10.1093/bioinformatics/btm009 17237039 PMC2387122

[15] Meyer F, Goesmann A, McHardy AC, Bartels D, Bekel T, Clausen J, GenDB–an open source genome annotation system for prokaryote genomes. Nucleic Acids Res. 2003;31:2187–95. 10.1093/nar/gkg312 12682369 PMC153740

[16] Darling AE, Mau B, Perna NT. Progressive Mauve: multiple genome alignment with gene gain, loss and rearrangement. PLoS ONE. 2010;5:e11147. 10.1371/journal.pone.0011147 20593022 PMC2892488

[17] Guindon S, Delsuc F, Dufayard JF, Gascuel O. Estimating maximum likelihood phylogenies with PhyML. Methods Mol Biol. 2009;537:113–37. 10.1007/978-1-59745-251-9_6 19378142

[18] Thompson JD, Higgins DG, Gibson TJ. CLUSTAL W: improving the sensitivity of progressive multiple sequence alignment through sequence weighting, position-specific gap penalties and weight matrix choice. Nucleic Acids Res. 1994;22:4673–80. 10.1093/nar/22.22.4673 7984417 PMC308517

[19] Anisimova M, Gascuel O. Approximate likelihood-ratio test for branches: a fast, accurate, and powerful alternative. Syst Biol. 2006;55:539–52. 10.1080/10635150600755453 16785212

[20] Petkau A, Stuart-Edwards M, Stothard P, Van Domselaar G. Interactive microbial genome visualization with GView. Bioinformatics. 2010;26:3125–6. 10.1093/bioinformatics/btq588 20956244 PMC2995121

[21] Mason PR. Zimbabwe experiences the worst epidemic of cholera in Africa. J Infect Dev Ctries. 2009;3:148–51. 10.3855/jidc.62 19755746

[22] Chun J, Grim CJ, Hasan NA, Je HL, Seon YC, Haley BJ, Comparative genomics reveals mechanism for short-term and long-term clonal transitions in pandemic *Vibrio cholerae.* Proc Natl Acad Sci U S A. 2009;106:15442–7. 10.1073/pnas.0907787106 19720995 PMC2741270

[23] Olsvik O, Wahlberg J, Petterson B, Uhlen M, Popovic T, Wachsmuth IK, Use of automated sequencing of polymerase chain reaction–generated amplicons to identify three types of cholera toxin subunit B in *Vibrio cholerae* O1 strains. J Clin Microbiol. 1993;31:22–5. 7678018 10.1128/jcm.31.1.22-25.1993 PMC262614

[24] Gilmour MW, Graham M, Van Domselaar G, Tyler S, Kent H, Trout-Yakel KM, High-throughput genome sequencing of two *Listeria monocytogenes* clinical isolates during a large foodborne outbreak. BMC Genomics. 2010;11:120. 20167121 10.1186/1471-2164-11-120 PMC2834635

[25] Gardy JL, Johnston JC, Ho Sui SJ, Cook VJ, Shah L, Brodkin E, Whole genome sequencing and social network analysis of a tuberculosis outbreak. N Engl J Med. 2011;364:730–9. 10.1056/NEJMoa1003176 21345102

[26] Taboada EN, van Belkum A, Yuki N, Acedillo RR, Godschalk PC, Koga M, Comparative genomic analysis of *Campylobacter jejuni* associated with Guillain-Barré and Miller Fisher syndromes: neuropathogenic and enteritis-associated isolates can share high levels of genomic similarity. BMC Genomics. 2007;8:359. 10.1186/1471-2164-8-359 17919333 PMC2174954

[27] Cho YJ, Yi H, Lee JH, Kim DW, Chun J. Genomic evolution of *Vibrio cholerae.* Curr Opin Microbiol. 2010;13:646–51. 10.1016/j.mib.2010.08.007 20851041

[28] Croucher NJ, Harris SR, Fraser C, Quail MA, Burton J, van der Linden M, Rapid pneumococcal evolution in response to clinical interventions. Science. 2011;331:430–4. 10.1126/science.1198545 21273480 PMC3648787

[29] Goel AK, Jain M, Kumar P, Bhadauria S, Kmboj DV, Singh L. A new variant of *Vibrio cholerae* O1 El Tor causing cholera in India. J Infect. 2008;57:280–1. 10.1016/j.jinf.2008.06.015 18657323

[30] Quilici ML, Massenet D, Gake B, Bwalki B, Olson DM. *Vibrio cholerae* O1 variant with reduced susceptibility to ciprofloxacin, western Africa. Emerg Infect Dis. 2010;16:1804–5. 21029554 10.3201/eid1611.100568 PMC3294521

