# Supplementary material for: Comparative Genomics of Vibrio cholerae from Haiti, Asia, and Africa
Source: Emerg Infect Dis. 2011 Nov;17(11):2113–21. doi: 10.3201/eid1711.110794 (PMC3310578; doi:10.3201/eid1711.110794)
Supplement: Technical Appendix 2 — Contig data, reconstructed core genome phylogeny of Vibrio cholerae figures. [file 11-0794-Techapp2_3p.pdf]

Article DOI: http://dx.doi.org/10.3201/eid1711.110794

Comparative Genomics of *Vibrio cholerae* from Haiti, Asia, and Africa

Technical Appendix 2

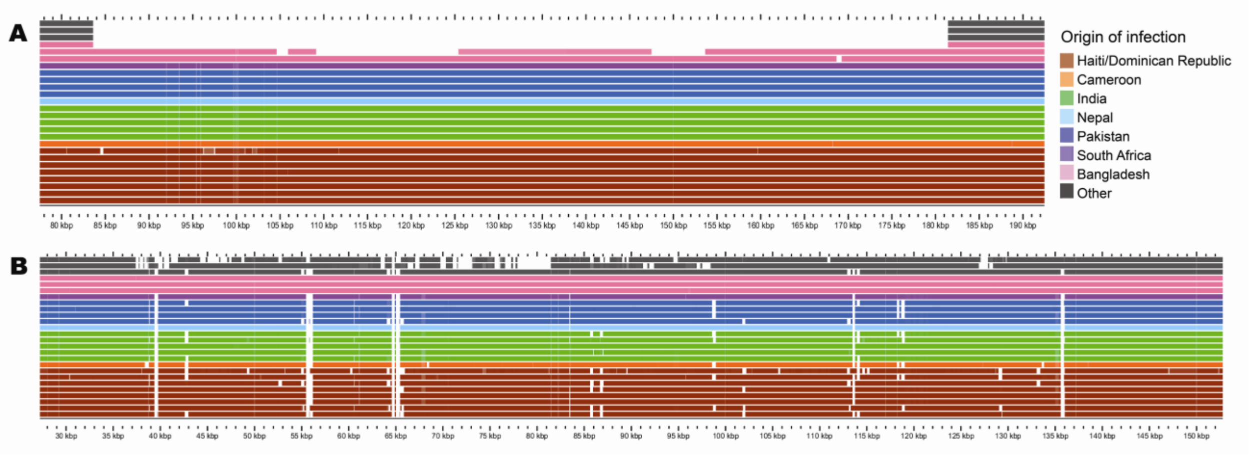

Technical Appendix 2 Figure 1. Contig data (>500 bp) mapped to A) integrative conjugative element belonging to the SXT/R391 family and B) superintegron regions of Haiti outbreak *Vibrio cholerae* isolate 2010EL-1786, fall 2010.

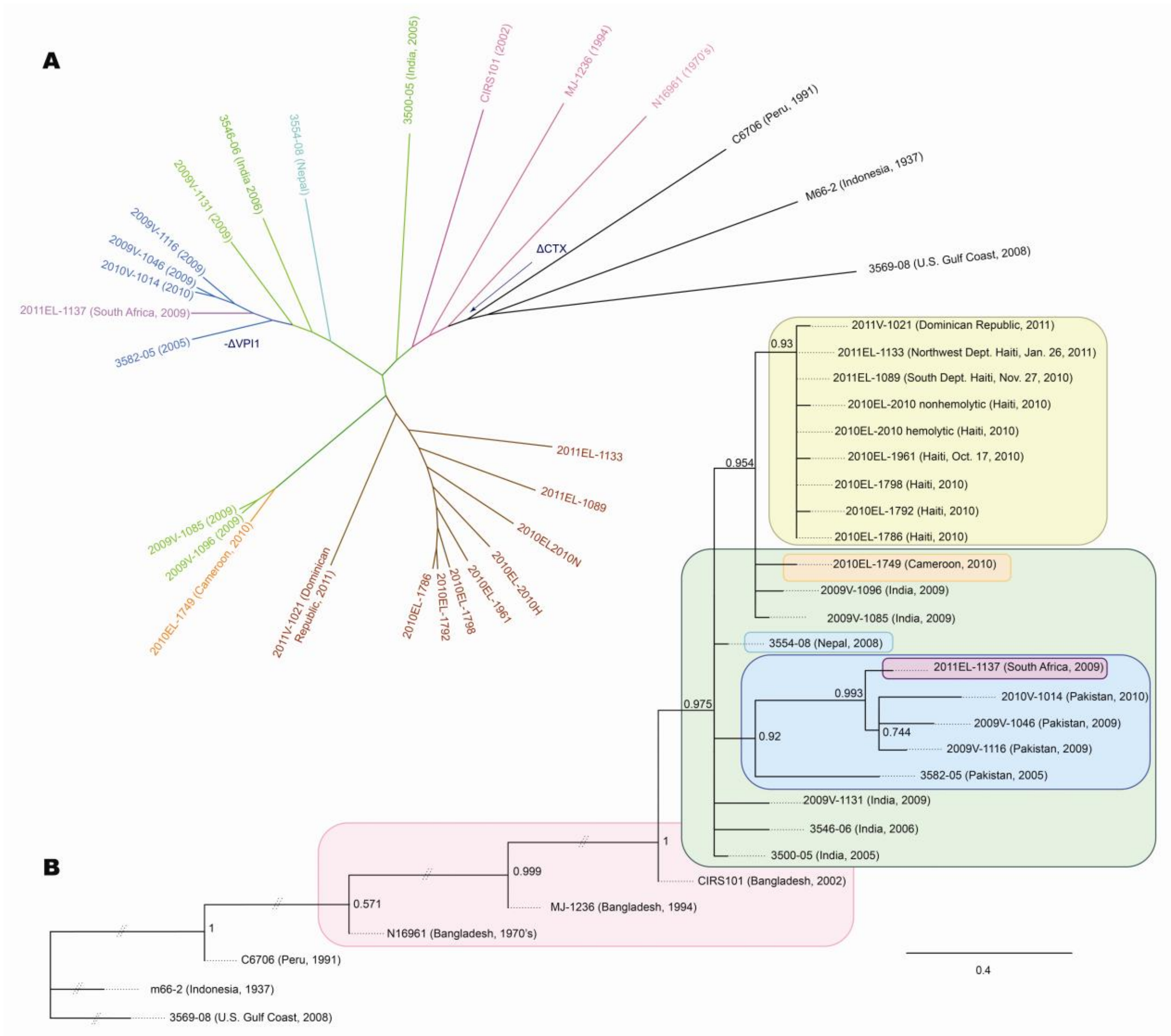

C

	Haiti/Dominican Republic									India		Cameroo	India	Nepal	India		South Africa	Pakistan				Bangladesh				
Isolate	2010EL-1786	2010EL-1798	2010EL-2010H	2011EL-1089	2011EL-1133	2010EL-1792	2010EL-1961	2010EL-2010N	2011V-1021	2009V-1085	2009V-1096	2010EL-1749	3500-05	3554-08	2009V-1131	3546-06	2011EL-1137	2010V-1014	2009V-1046	2009V-1116	3582-05	CIRS101	MJ1236	N16961	C6706	M66-2
2010EL-1786																										
2010EL-1798	0																									
2010EL-2010H	0	0																								
2011EL-1089	0	0	0																							
2011EL-1133	0	0	0	0																						
2010EL-1792	1	1	1	1	1																					
2010EL-1961	1	1	1	1	1	2																				
2010EL-2010N	1	1	1	1	1	2	2																			
2011V-1021	1	1	1	1	1	2	2	2																		
2009V-1085	4	4	4	4	4	5	5	5	5																	
2009V-1096	5	5	5	5	5	6	6	6	6	3																
2010EL-1749	6	6	6	6	6	7	7	7	7	4	5															
3500-05	7	7	7	7	7	8	8	8	8	5	6	7														
3554-08	7	7	7	7	7	8	8	8	8	5	6	7	2													
2009V-1131	10	10	10	10	10	11	11	11	11	8	9	10	5	5												
3546-06	10	10	10	10	10	11	11	11	11	8	9	10	5	5	8											
2011EL-1137	19	19	19	19	19	20	20	20	20	17	18	19	14	14	17	17										
2010V-1014	24	24	24	24	24	25	25	25	25	22	23	24	19	19	22	22	9									
2009V-1046	22	22	22	22	22	23	23	23	23	20	21	22	17	17	20	20	7	10								
2009V-1116	20	20	20	20	20	21	21	21	21	18	19	20	15	15	17	18	5	8	6							
3582-05	18	18	18	18	18	19	19	19	19	16	17	18	13	13	16	16	19	24	22	20						
CIRS101	13	13	13	13	13	14	14	14	14	11	12	13	8	8	11	11	20	25	23	21	19					
MJ1236	47	47	47	47	47	48	48	48	48	45	46	47	42	42	45	45	54	59	57	55	53	38				
N16961	99	99	99	99	99	100	100	100	100	97	98	99	94	94	97	97	106	111	109	107	105	90	76			
C6706	102	102	102	102	102	103	103	103	103	100	101	102	97	97	100	100	109	114	112	110	108	93	79	87		
M66-2	1996	1996	1996	1996	1996	1997	1997	1997	1997	1994	1995	1996	1991	1991	1994	1994	2003	2008	2006	2004	2002	1987	1974	1982	1971	
3569-08	3349	3349	3349	3349	3349	3350	3350	3350	3350	3347	3348	3349	3344	3344	3347	3347	3356	3361	3359	3357	3355	3340	3326	3334	3323	2975

Technical Appendix 2 Figure 2. Reconstructed core genome phylogeny of *Vibrio cholerae* based on 4,376 high-quality, single-nucleotide polymorphisms among 632 fully sequenced orthologous genes with >97% homology between alleles. A) Proportional cladogram. Dept., department. B) Maximum-likelihood phylogenetic tree with node confidences expressed as approximate likelihood ratios. C) Pair-wise single-nucleotide polymorphism matrix used to calculate phylogenetic distances and generate trees in A and B. Scale bar indicates nucleotide substitutions per site.

A

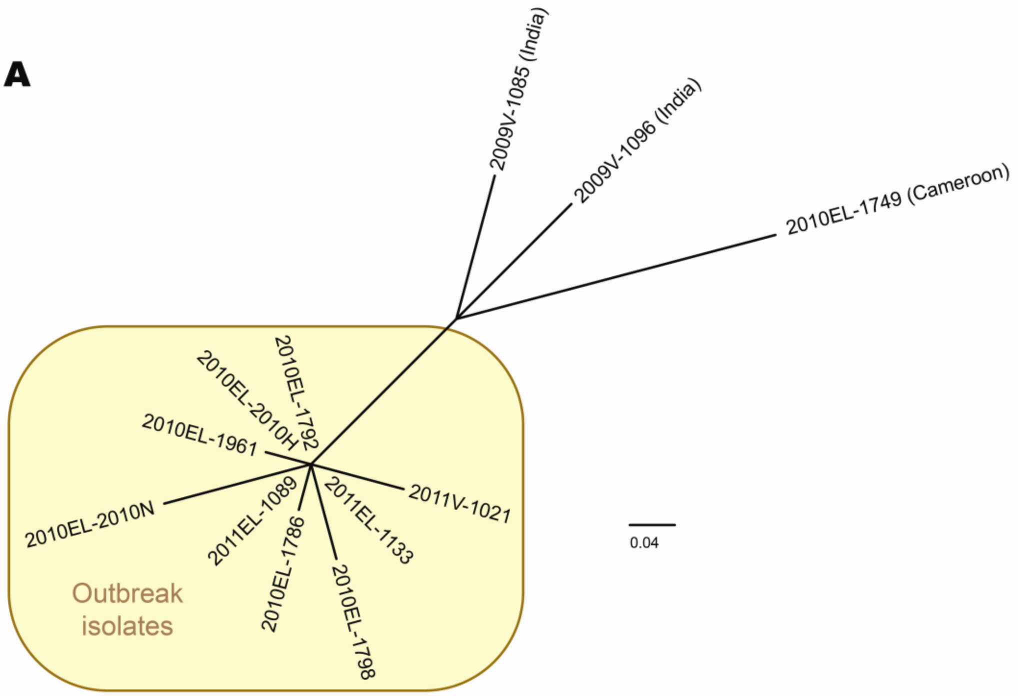

B

Isolate	2010EL-1786	2010EL-1792	2010EL-2010H	2011EL-1089	2011EL-1133	2010EL-1961	2010EL-1798	2011V-1021	2010EL-2010N	2009V-1085	2009V-1096
2010EL-1786											
2010EL-1792	1										
2010EL-2010H	1	0									
2011EL-1089	1	0	0								
2011EL-1133	1	0	0	0							
2010EL-1961	2	1	1	1	1						
2010EL-1798	3	2	2	2	2	3					
2011V-1021	3	2	2	2	2	3	4				
2010EL-2010N	4	3	3	3	3	4	5	5			
2009V-1085	8	7	7	7	7	8	9	9	10		
2009V-1096	8	7	7	7	7	8	9	9	10	6	
2010EL-1749	11	10	10	10	10	11	12	12	13	9	9

Technical Appendix 2 Figure 3. Reconstructed core genome phylogeny of *Vibrio cholerae* based on 25 high-quality, single-nucleotide polymorphisms among 24 fully sequenced orthologous genes with >97% homology between alleles. A) Proportional cladogram. Scale bar indicates nucleotide substitutions per site. B) Pair-wise single-nucleotide polymorphism matrix used to calculate phylogenetic distances and generate tree in panel A.
